# Potentiation of Nerve Growth Factor-Induced Neurite Outgrowth by Fluvoxamine: Role of Sigma-1 Receptors, IP_3_ Receptors and Cellular Signaling Pathways

**DOI:** 10.1371/journal.pone.0002558

**Published:** 2008-07-02

**Authors:** Tomoko Nishimura, Tamaki Ishima, Masaomi Iyo, Kenji Hashimoto

**Affiliations:** 1 Division of Clinical Neuroscience, Chiba University Center for Forensic Mental Health, Chiba, Japan; 2 Division of Medical Treatment and Rehabilitation, Chiba University Center for Forensic Mental Health, Chiba, Japan; 3 Department of Psychiatry, Chiba University Graduate School of Medicine, Chiba, Japan; James Cook University, Australia

## Abstract

**Background:**

Selective serotonin reuptake inhibitors (SSRIs) have been widely used and are a major therapeutic advance in psychopharmacology. However, their pharmacology is quite heterogeneous. The SSRI fluvoxamine, with sigma-1 receptor agonism, is shown to potentiate nerve-growth factor (NGF)-induced neurite outgrowth in PC 12 cells. However, the precise cellular and molecular mechanisms underlying potentiation by fluvoxamine are not fully understood. In this study, we examined the roles of cellular signaling pathways in the potentiation of NGF-induced neurite outgrowth by fluvoxamine and sigma-1 receptor agonists.

**Methods and Findings:**

The effects of three SSRIs (fluvoxamine, sertraline, paroxetine) and three sigma-1 receptor agonists (SA4503, 4-phenyl-1-(4-phenylbutyl) piperidine (PPBP), and dehydroepiandrosterone (DHEA)-sulfate) on NGF-induced neurite outgrowth in PC12 cells were examined. Also examined were the effects of the sigma-1 receptor antagonist NE-100, inositol 1,4,5-triphosphate (IP_3_) receptor antagonist, and specific inhibitors of signaling pathways in the potentiation of NGF-induced neurite outgrowth by selective sigma-1 receptor agonist SA4503. Fluvoxamine (but not sertraline or paroxetine) and the sigma-1 receptor agonists SA4503, PPBP, and DHEA-sulfate significantly potentiated NGF-induced neurite outgrowth in PC12 cells in a concentration-dependent manner. The potentiation by fluvoxamine and the three sigma-1 receptor agonists was blocked by co-administration of the selective sigma-1 receptor antagonist NE-100, suggesting that sigma-1 receptors play a role in blocking the enhancement of NGF-induced neurite outgrowth. Moreover, the potentiation by SA4503 was blocked by co-administration of the IP_3_ receptor antagonist xestospongin C. In addition, the specific inhibitors of phospholipase C (PLC-γ), phosphatidylinositol 3-kinase (PI3K), p38MAPK, c-Jun N-terminal kinase (JNK), and the Ras/Raf/mitogen-activated protein kinase (MAPK) signaling pathways blocked the potentiation of NGF-induced neurite outgrowth by SA4503.

**Conclusion:**

These findings suggest that stimulation of sigma-1 receptors and subsequent interaction with IP_3_ receptors, PLC-γ, PI3K, p38MAPK, JNK, and the Ras/Raf/MAPK signaling pathways are involved in the mechanisms of action of sigma-1 receptor agonists such as fluvoxamine and SA4503.

## Introduction

Selective serotonin (5-HT; 5-hydroxytryptamine) reuptake inhibitors (SSRIs) have emerged as a major therapeutic advance in psychopharmacology. SSRIs are the treatment of choice for many indications, including major depressive disorder, dysthymia, panic disorder, obsessive-compulsive disorder, eating disorders, and premenstrual dysphoric disorder. In contrast, it is well known that their pharmacology is quite heterogeneous, although all of them block 5-HT transporters, thus increasing 5-HT levels throughout the central nervous system (CNS) [1]–[9].

Accumulating evidence suggests that sigma-1 receptors, which are intracellular endoplasmic reticulum (ER) proteins, are involved in both the neuroplasticity and pathophysiology of neuropsychiatric diseases such as major depressive disorder, anxiety, schizophrenia, and Alzheimer's disease [10]–[18]. Previously, we reported that some SSRIs possess high to moderate affinities for sigma-1 receptors in the rat brain. The rank order of SSRIs affinities for sigma-1 receptors is fluvoxamine (K_i_ = 36 nM)>sertraline (K_i_ = 57 nM)>>paroxetine (K_i_ = 1893 nM) [19]. Recently, we reported that fluvoxamine, but not paroxetine, significantly ameliorated cognitive deficits in mice after repeated phencyclidine administration, and that the effects of fluvoxamine were antagonized by co-administration of the selective sigma-1 receptor antagonist NE-100 [20], suggesting that sigma-1 receptors are involved in the mechanism of action of fluvoxamine [9]. Interestingly, it has been demonstrated that sigma-1 receptor agonists including fluvoxamine could potentiate nerve growth factor (NGF)-induced neurite outgrowth in PC12 cells, and that NE-100 blocked the potentiation by sigma-1 receptor agonists, suggesting sigma-1 receptors are involved in neuroplasticity [21]. However, the precise cellular mechanisms underlying the potentiation by sigma-1 receptor agonists are not fully understood [13], [21].

It is therefore of great interest to study the precise cellular mechanisms underlying the enhancement by fluvoxamine on NGF-induced neurite sprouting in PC12 cells. In the present study, we examined the effects of three SSRIs (fluvoxamine, sertraline, paroxetine), as well as the effects of a sigma-1 receptor agonist (4-phenyl-1-(4-phenylbutyl) piperidine (PPBP), dehydroepiandrosterone-sulphate (DHEA)-sulfate) [9], [22]–[28] and the selective sigma-1 receptor agonist SA4503 [29], [30], on NGF-induced neurite outgrowth in PC12 cells. Furthermore, it is also known that sigma-1 receptors have been shown to interact with IP_3_ receptors (17,18). Therefore, we examined the effects of NE-100 and xestospongin C (a selective inositol 1,4,5-triphosphate (IP_3_) receptor antagonist) [31] in order to investigate the roles of sigma-1 receptors and IP_3_ receptors in the mechanisms underlying the enhancement of NGF-induced neurite outgrowth by SA4503. Moreover, we examined the effects of specific inhibitors of several cellular signaling targets on the enhancement of NGF-induced neurite outgrowth by SA4503, since several signal transduction molecules have been implicated in NGF-induced neurite outgrowth [32].

## Materials and Methods

### Drugs

The drugs were obtained from the following sources: fluvoxamine maleate (Solvay Seiyaku K.K., Tokyo, Japan); paroxetine hydrochloride, dehydroepiandosterone-sulfate (DHEA-sulfate), LY294002 (Sigma-Aldrich, St Louis, MO, USA); sertraline (Toronto Research Chemicals Inc., North York, ON, Canada); SA4503 (M's Science Corporation, Kobe, Japan); NGF (Promega, Madison, WI); xestospongin C, lovastatin, PD98059, GW5074, SB203580, MEK 1/2 inhibitor (SL327), and SP600125 (Calbiochem-Novabiochem, San Diego, CA). The selective sigma-1 receptor antagonists NE-100 and 4-phenyl-1-(4-phenylbutyl) piperidine (PPBP) were synthesized in our laboratory. Other drugs were purchased from commercial sources.

### Cell culture

**Figure 1:**
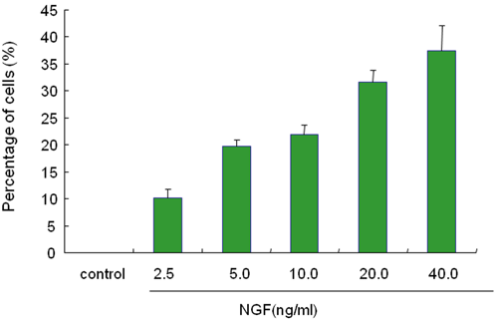
Effects of NGF on neurite outgrowth in PC12 cells. Incubation of NGF (0, 2.5, 5.0, 10.0, 20.0 or 40.0 ng/ml) caused neurite outgrowth in PC12 cells in a concentration dependent manner. The data were shown as percentage of the number of cells with neurite outgrowth in the number of total cells. The data show the mean±S.E.M (n = 8).

PC12 sells (RIKEN Cell Bank, Tsukuba, Japan) were cultured at 37°C, 5% CO_2_ with Dulbecco's modified Eagle's medium (DMEM) supplemented with 5% heat-inactivated fetal bovine serum (FBS), 10% heat-inactivated horse serum, and 1% penicillin. The medium was changed two or three times a week. PC12 cells were plated onto 24-well tissue culture plates coated with poly-D-lysine/laminin. Cells were plated at relatively low density (0.25×10^4^ cells/cm^2^) in DMEM medium containing 0.5% FBS, 1% penicillin streptomycin. Medium containing a minimal level of serum (0.5% FBS) was used, since serum is known to contain steroid hormones that bind to sigma-1 receptors [21]. First, we examined the optimal concentration of NGF for NGF-induced neurite outgrowth in PC12 cells. NGF (2.5, 5, 10, 20, 40 ng/ml) increased the number of cells with neurite outgrowth in PC12 cells in a concentration-dependent manner (**Figure 1**). In the subsequent studies, 2.5 ng/ml of NGF was used to study the potentiating effects of sigma-1 receptor agonists on NGF-induced neurite outgrowth. Twenty-four hours after plating, the medium was replaced with DMEM medium containing 0.5% FBS and 1% penicillin streptomycin with NGF (2.5 ng/ml) with or without several drugs.

### Quantification of neurite sprouting

Five days after incubation with NGF (2.5 ng/ml) with or without the several drugs, morphometric analysis was performed on digitized images of live cells taken under phase-contrast illumination with an inverted microscope linked to a camera. Images of three fields per well were taken, with an average of 100 cells per field. Differentiated cells were counted by visual examination of the field; only cells that had at least one neurite with a length equal to the cell body diameter were counted, and were then expressed as a percentage of the total cells in the field. The counting was performed in a blinded manner.

### Immunocytochemistry

Cells were fixed for 30 min at room temperature with 4% paraformaldehyde then permeabilized with 0.2% Triton and blocked with 1.5% normal goat serum, 0.1% bovine serum albumin (BSA) in 0.1 M phosphate-buffer saline for 1 h to reduce nonspecific binding. Cells were incubated overnight at 4°C with anti-microtubule-associated protein 2 (MAP-2) antibodies (1∶1000 dilution in blocking solution, Chemicon International, Temecula, CA, USA). The immunolabeling was visualized with secondary antibodies conjugated to Alexa-488 (1∶1000; Invitrogen, Carlsbad, CA, USA). MAP-2 immuncytochemistry was visualized with a fluorescence microscope (Axiovert 200, Carl Zeiss, Oberkocken, Germany).

### Statistical analysis

Data are expressed as means±S.E.M. Statistical analysis was performed by using one-way analysis of variance (ANOVA) and the *post hoc* Bonferroni/Dunn test. *P* values less than 0.05 were considered statistically significant.

## Results

### Effects of SSRIs on NGF-induced neurite outgrowth

**Figure 2:**
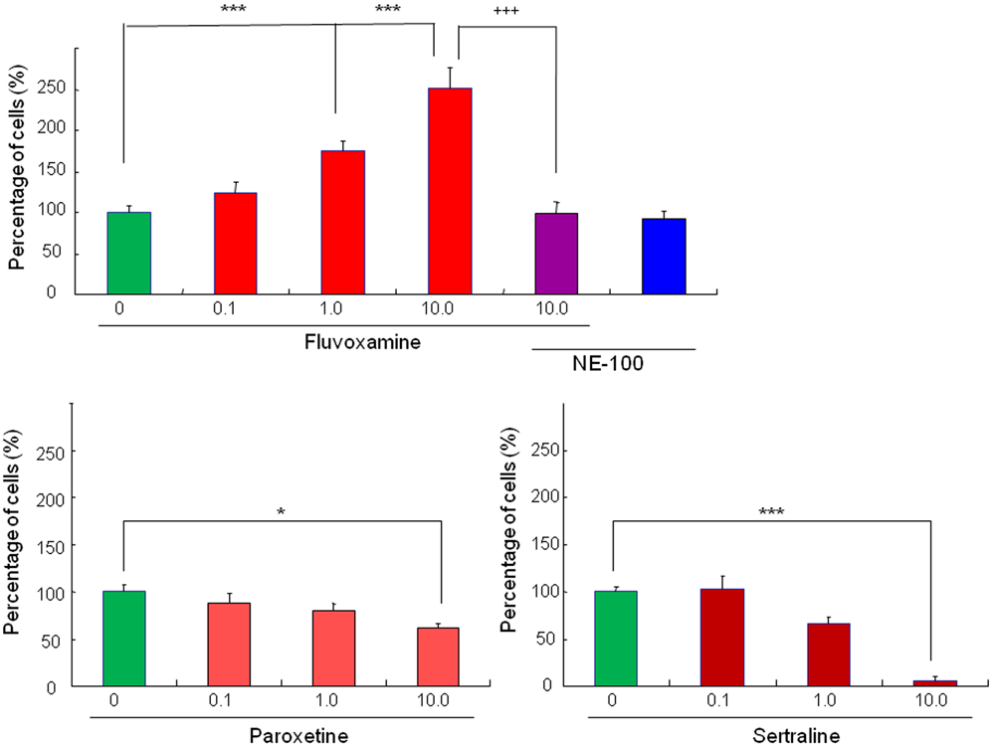
Effects of three SSRIs (fluvoxamine, sertraline, paroxetine) on NGF-induced neurite outgrowth in PC12 cells. Fluvoxamine (The data show the mean±S.E.M (n = 6)), paroxetine (The data show the mean±S.E.M (n = 6)), sertraline (The data show the mean±S.E.M (n = 6)). Fluvoxamine (but not sertraline or paroxetine) significantly potentiated NGF-induced neurite outgrowth in PC12 cells in a concentration-dependent manner. The potentiation by fluvoxamine was blocked by co-administration of the selective sigma-1 receptor antagonist NE-100 (1.0 µM). Number is the concentration (µM) of drugs. *P<0.05, ***P<0.001 as compared with control (NGF alone group). ^+++^P<0.001 as compared with fluvoxamine (10.0 µM) plus NE-100 group.

Fluvoxamine (0.1, 1.0, or 10 µM) significantly increased the number of cells with neurite outgrowth by NGF (2.5 ng/ml) in PC12 cells, in a concentration-dependent manner (**Figure 2** **and** **4**). In contrast, sertraline (0.1, 1.0, or 10 µM) and paroxetine (0.1, 1.0, or 10 µM) did not increase the number of cells with neurite outgrowth by NGF (2.5 ng/ml). A higher concentration (10 µM) of paroxetine and sertraline significantly decreased the number of cells with NGF-induced neurite outgrowth, suggesting the cellular toxicity of these drugs in PC12 cells (**Figure 2**).

### Role of sigma-1 receptors in the potentiation of NGF-induced neurite outgrowth by fluvoxamine and SA4503

**Figure 3:**
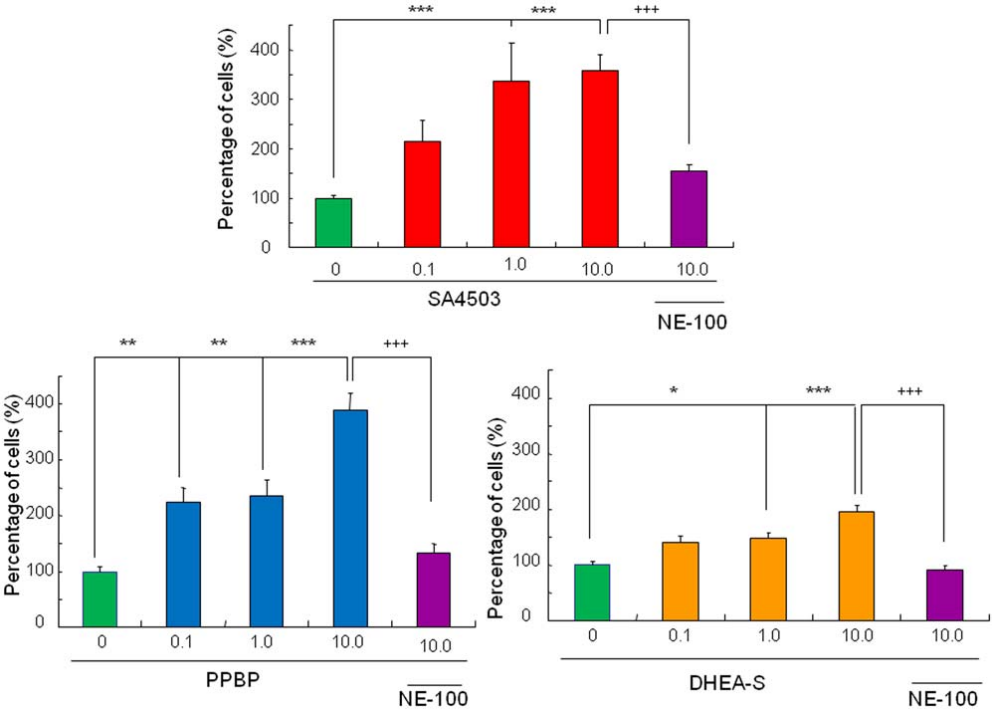
Effects of three sigma-1 receptor agonists (SA4503, PPBP, DHEA-sulfate) on NGF-induced neurite outgrowth in PC12 cells. SA4503 (The data show the mean±S.E.M (n = 5–15)), PPBP (The data show the mean±S.E.M (n = 8)), DHEA-sulfate (The data show the mean±S.E.M (n = 8)). These drugs (0.1, 1.0 or 10.0 µM) significantly potentiated NGF-induced neurite outgrowth in PC12 cells in a concentration-dependent manner. The potentiation by these drugs was significantly blocked by co-administration of the selective sigma-1 receptor antagonist NE-100 (1.0 µM). Number is the concentration (µM) of drugs. *P<0.05, **P<0.01, ***P<0.001 as compared with control (NGF alone group). ^+++^P<0.001 as compared with drugs (10 µM) plus NE-100 group.

The selective and potent sigma-1 receptor agonist SA4503 (0.01, 0.1, or 1.0 µM) significantly increased the number of cells with neurite outgrowth by NGF (2.5 ng/ml) in PC12 cells, in a concentration-dependent manner (**Figure 3** **and** **4**). In addition, other sigma-1 receptor agonists–PPBP (0.1, 1.0, or 10 µM) [22]–[26] and DHEA-sulphate (0.1, 1.0, or 10 µM) [9], [27], [28]–significantly increased the number of such cells, also in a concentration-dependent manner (**Figure 3**). In the absence of NGF, sigma-1 receptor agonists did not produce neurite outgrowth in PC12 cells (data not shown).

**Figure 4:**
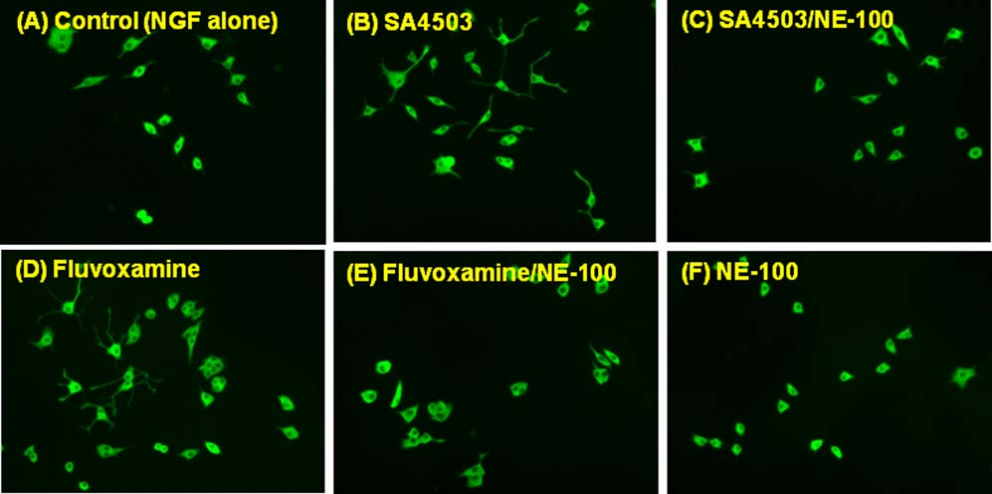
Representative photographs of MAP-2 immunocytochemistry in PC12 cells. (A) Control (NGF (2.5 ng/ml) alone) (B) NGF+SA4503 (1.0 µM), (C) NGF+SA4503 (1.0 µM)/NE-100 (1.0 µM), (D) NGF+Fluvoxamine (10.0 µM), (E) NGF+Fluvoxamine (10.0 µM)/NE-100 (1.0 µM), (F) NE-100 (1.0 µM). Incubation of SA4503 (1.0 µM) or fluvoxamine (10 µM) potentiated NGF-induced neurite outgrowth in PC12 cells. The potentiating effects of SA4503 or fluvoxamine on the NGF-induced neurite outgrowth were antagonized by co-administration of NE-100 (1.0 µM). Furthermore, NE-100 (1.0 µM) alone did not alter NGF-induced neurite outgrowth.

To investigate the role of sigma-1 receptors, we examined the effects of NE-100 (a selective sigma-1 receptor antagonist) on the potentiation of NGF-induced neurite outgrowth by fluvoxamine, SA4503, PPBP, and DHEA-sulphate. Co-administration of NE-100 (1.0 µM) significantly blocked such potentiation by fluvoxamine (10 µM), SA4503 (1.0 µM), PPBP (10 µM), or DHEA-sulphate (10 µM) (**Figure 2** **and** **3**). Furthermore, administration of NE-100 (1.0 µM) alone did not alter NGF-induced neurite outgrowth in PC12 cells (**Figure 2**).

### Role of IP_3_ receptors in the potentiation of NGF-induced neurite outgrowth by SA4503

**Figure 5:**
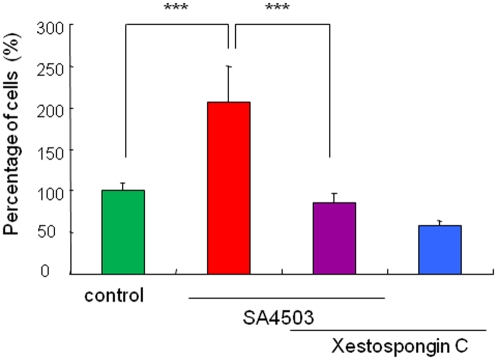
Effects of the IP3 receptor antagonist xestospongin C on potentiation of NGF-induced neurite outgrowth by SA4503. The potentiating effects of SA4503 (1.0 µM) on the NGF-induced neurite outgrowth were antagonized by co-administration of xestospongin C (1.0 µM). Furthermore, xestospongin C (1.0 µM) alone did not alter NGF-induced neurite outgrowth. The data show the mean±SEM (n = 12). ***p<0.001.

Sigma-1 receptors have been shown to interact with IP_3_ receptors (17,18,33–35). To investigate the role of IP_3_ receptors in the effects of SA4503 on NGF-induced neurite outgrowth, we examined the effects of xestospongin C (a selective, reversible, and membrane-permeable inhibitor of IP_3_ receptors) [31] on the effects of SA4503 on NGF-induced neurite outgrowth. Co-administration of xestospongin C (1.0 µM) significantly blocked the potentiation of NGF-induced neurite outgrowth by SA4503 (1.0 µM) (**Figure 5**). Furthermore, administration of xestospongin C (1.0 µM) alone did not alter NGF-induced neurite outgrowth in PC12 cells (**Figure 5**).

### Role of signaling molecules proximal to TrkA in the potentiation of NGF-induced neurite outgrowth by SA4503

**Figure 6:**
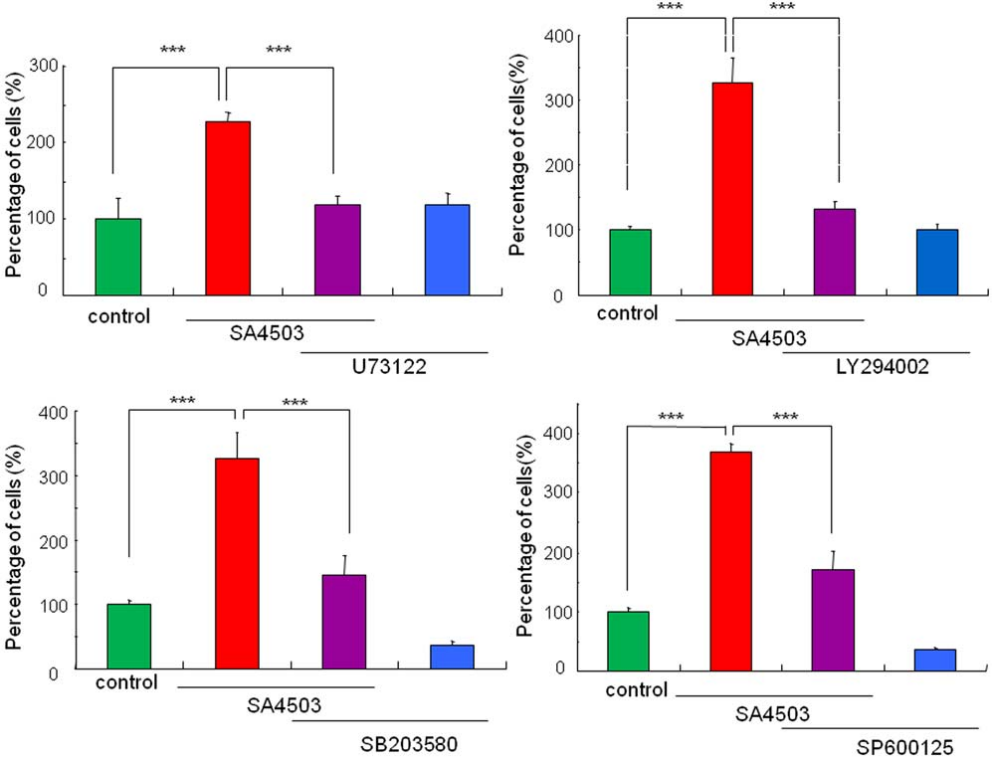
Effects of the specific inhibitors of PLC-γ, PI3K, p38MAPK, and JNK on potentiation of NGF-induced neurite outgrowth by SA4503. The potentiating effects of SA4503 (1.0 µM) on the NGF-induced neurite outgrowth were antagonized by co-administration of the PLC-γ inhibitor (U73122; 1.0 µM), the PI3K inhibitor (LY294002; 1.0 µM), the p38MAPK inhibitor (SB203580; 1.0 µM), and the JNK inhibitor (SP600125; 1.0 µM). The data show the mean±SEM (n = 4–12). ***p<0.001.

Next, we examined the effects of the specific inhibitors of PLC-γ, PI3K, p38 MAPK, and c-Jun N-terminal kinase (JNK), since these signaling molecules are activated upon the addition of NGF [36]. The PLC-γ inhibitor (U73122; 1.0 µM), PI3K inhibitor (LY294002; 1.0 µM), p38 MAPK inhibitor (SB203580; 1.0 µM), and JNK inhibitor (SP600125; 1.0 µM) significantly blocked the potentiation of NGF-induced neurite outgrowth by SA4503 (1.0 µM) (**Figure 6**). In contrast, these inhibitors alone did not alter NGF-induced neurite outgrowth in PC12 cells (**Figure 6**).

### Role of Raf/Ras/ERK/MAPK pathway in the potentiation of NGF-induced neurite outgrowth by SA4503

**Figure 7:**
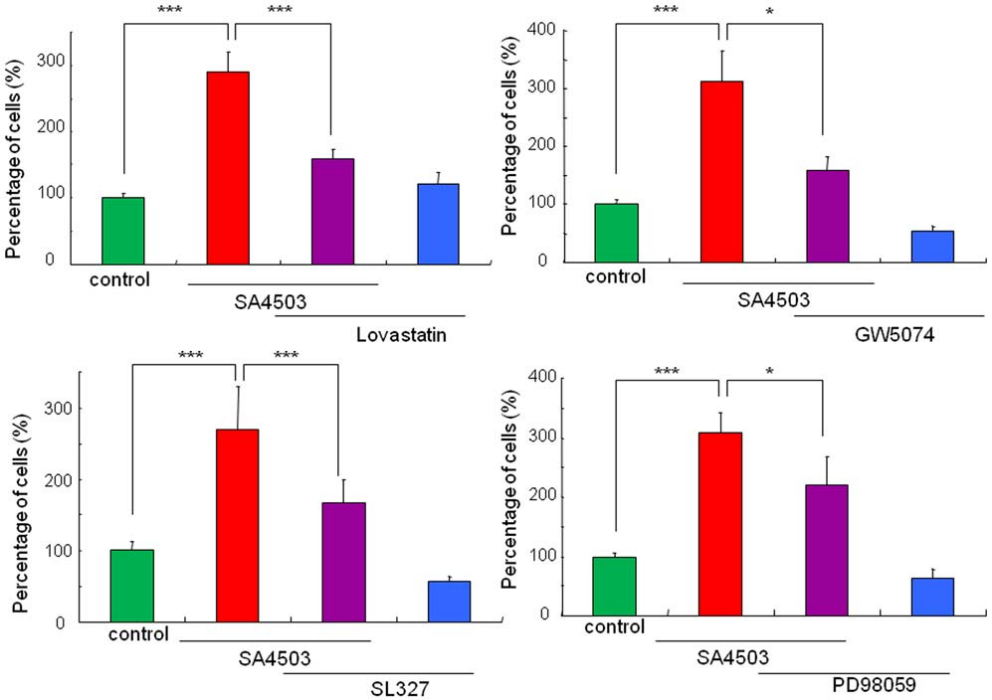
Effects of the specific inhibitors of Ras, Raf, MEK1/2, and MAPK on potentiation of NGF-induced neurite outgrowth by SA4503. The potentiating effects of SA4503 (1.0 µM) on the NGF-induced neurite outgrowth were antagonized by co-administration of the Ras inhibitor (GW5074; 1.0 µM), the Raf inhibitor (lovastatin; 1.0 µM), the MEK1/2 inhibitor (MEK1/2 inhibitor; 1.0 µM), and the MAPK inhibitor (PD98059; 1.0 µM). The data show the mean±SEM (n = 4 or 8). *P<0.05, ***p<0.001.

The Raf/Ras/ERK/MAPK pathway is known to be involved in NGF-induced outgrowth [32]. Therefore, we examined the effects of this pathway's specific inhibitors. The Ras inhibitor (GW5074; 1.0 µM), Raf inhibitor (lovastatin; 1.0 µM), MEK1/2 inhibitor (SL327; 1.0 µM), and MAPK inhibitor (PD98059; 1.0 µM) significantly blocked the potentiation of NGF-induced neurite outgrowth by SA4503 (1.0 µM) (**Figure 7**). In contrast, these inhibitors alone did not alter NGF-induced neurite outgrowth in PC12 cells (**Figure 7**).

## Discussion

In the present study, we found that fluvoxamine (but not sertraline or paroxetine) and the sigma-1 receptor agonists (SA4503, PPBP, DHEA-sulfate) could potentiate NGF-induced neurite outgrowth in PC12 cells, and that the effects of these drugs were blocked by co-incubation with the selective sigma-1 receptor antagonist NE-100. These findings suggest that agonism at sigma-1 receptors for these drugs is involved in the mechanisms underlying the drugs' potentiation of NGF-induced neurite outgrowth.

As shown in **Figure 1**, NGF increased the number of cells with neurite outgrowth in PC12, in a concentration dependent manner. To examine whether or not the NGF levels are altered by incubation with sigma-1 receptor agonists, we measured the levels of NGF in PC12 cells with or without sigma-1 receptor agonist SA4503. No differences of NGF levels were shown in the between control group and SA4503-treated group (data not shown). It is, therefore, unlikely that the potentaition of NGF-induced neurite outgrowth by sigma-1 receptor agonists might be due to increased levels of NGF.

Unlike fluvoxamine, sertraline, which has a moderate affinity for sigma-1 receptors, did not alter NGF-induced neurite outgrowth. The reasons underlying this discrepancy between these two SSRIs are currently unclear. One possibility may involve the difference in pharmacological actions (agonist vs. antagonist) between these SSRIs at sigma-1 receptors. Interestingly, we recently found that, in a novel object recognition test, phencyclidine-induced cognitive deficits could be significantly improved by subsequent subchronic (14 days) administration of fluvoxamine, but not of sertraline, suggesting that sigma-1 receptor agonism is involved in fluvoxamine's mechanism of action [9], Ishima et al., submitted. Taken together, these findings suggest that fluvoxamine and sertraline may function as an agonist and an antagonist at sigma-1 receptors, respectively, although further study is necessary. Another possibility may be that other pharmacological activities of sertraline mask the effects of sigma-1 receptor agonism. In this study, we also found that high concentration (10 µM) of paroxetine and sertraline, but not fluvoxamine, showed cytotoxicity in PC12 cells, suggesting that fluvoxamine may be a safe drug than paroxetine and sertraline.

Sigma-1 receptors have been shown to affect intracellular Ca^2+^ signaling, although the precise molecular and cellular mechanisms underlying this effect are unknown. Sigma-1 receptors bind to IP_3_ receptors in ER, and sigma-1 receptors regulate Ca^2+^ release from intracellular Ca^2+^ storage sites [12]. Very recently, Hayashi and Su [17] reported that sigma-1 receptors function as novel ligand-operated chaperones that specifically target mitochondrion-associated ER membrane. Furthermore, sigma-1 receptors form Ca^2+^-sensitive chaperone machinery with another chaperone, BiP, and prolong Ca^2+^ signaling from ER into mitochondria by stabilizing IP_3_ receptors at mitochondrion-associated ER membrane [17]. In this study, we found that the IP_3_ receptor antagonist xestospongin C significantly blocked the potentiation of NGF-induced neurite outgrowth by SA4503, suggesting the role of IP_3_ receptors on sigma-1 receptor-mediated potentiation of NGF-induced neurite outgrowth. Therefore, it is likely that stimulation at sigma-1 receptors by sigma-1 receptor agonists and subsequent interaction with IP_3_ receptors are involved in the mechanism underlying the potentiation of NGF-induced neurite outgrowth by sigma-1 receptor agonists.

**Figure 8:**
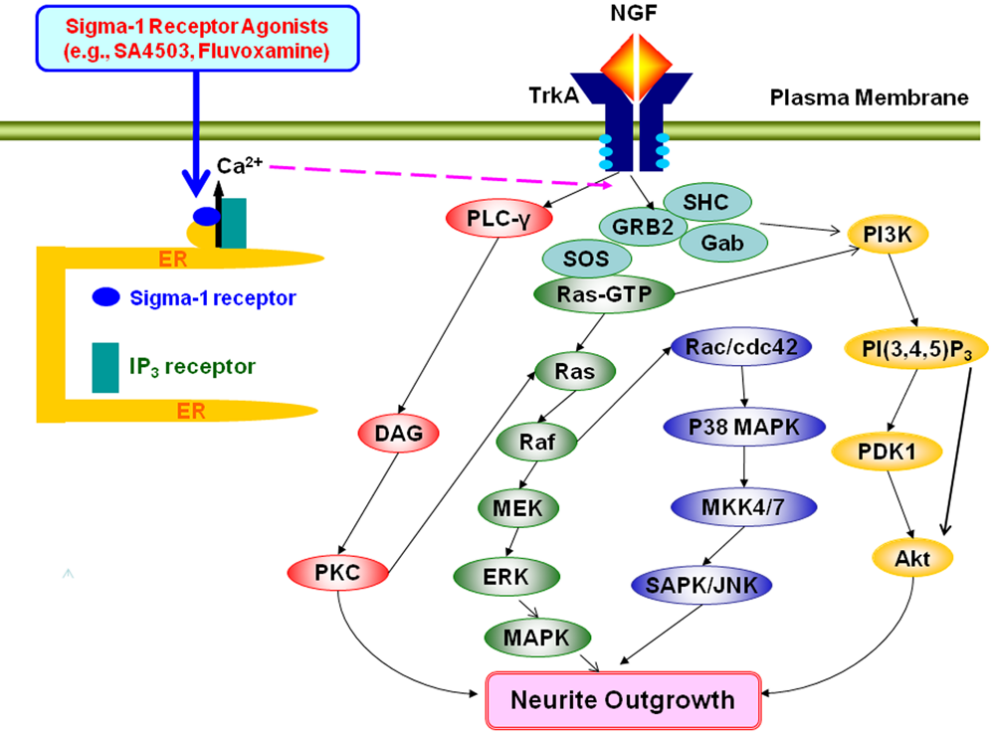
Proposed mechanism for potentiation of NGF-induced neurite outgrowth by sigma-1 receptor agonists. NGF binding to cell-surface TrkA receptor results in activated receptor complexes, which contain adaptors such as SHC (SH2-containing protein), GRB2 (growth-factor-receptor bound protein 2) and Gab (GRB2-associated binding) proteins. These proteins recruit the protein SOS, increasing Ras-guanosine triphosphate (Ras-GTP) levels by catalyzing nucleotide exchange on Ras. Ras also activates the PI3K-PDK1 (3-phosphoinosisitide-dependent protein kinase 1)- Akt pathway. The activation of TrkA by NGF leads to activation of multiple signaling pathways, including PLC-γ, PI3K, p38MAPK, and JNK signaling pathways and Ras/Raf/MEK/ERK/MAPK pathways. The activation of sigma-1 receptors on the endoplasmic reticulum (ER) by sigma-1 receptor agonists (e.g., SA4503 and fluvoxamine) might interact with IP3 receptors on the ER. Ca^2+^ released by the interaction between sigma-1 receptors and IP3 receptors may play a role in the potentiation of NGF-induced neurite outgrowth in PC12 cells. Subsequently, several signaling pathways are implicated in the potentiation of NGF-induced neurite outgrowth by sigma-1 receptor agonists.

NGF binds to the high-affinity tyrosine receptor TrkA, initiating several signaling pathways affecting both morphological and transcriptional targets [32], [36], [37]. The signaling molecules, including PLC-γ, PI3K, p38 MAPK, and JNK, are activated upon the addition of NGF [38]. PLC-γ catalyzes the hydrolysis of phosphatidylinositol-4,5-bisphosphate (PIP2) to diacylglycerol (DAG) and inositol triphosphate (IP3). DAG activates protein kinase C, and IP3 promotes transient release of Ca^2+^ from the ER [39]. The pathway via PLC-γ is responsible for NGF-induced cell differentiation [40] and neurite outgrowth [41]. Furthermore, stimulation of PI3K is reported to be involved in the promotion of neurite outgrowth in PC12 cells [42]. In this study, we found that the PLC-γ inhibitor U73122 and the PI3K inhibitor LY294002 significantly blocked the potentiation of NGF-induced neurite outgrowth by SA4503. Moreover, we found that both the p38MAPK inhibitor SB203580 and the JNK inhibitor SP600125 significantly blocked the potentiation of NGF-induced neurite outgrowth by SA4503. In addition, it is also interesting that neurite outgrowth induced by low concentration (2.5 ng/ml) of NGF was not blocked by these inihibitors, consistent with a previous report [43], [44], suggesting the existence of novel pathway(s) for NGF-induced neurite outgrowth. These findings suggest that the PLC-γ, PI3K, p38MAPK, and JNK signaling pathways are involved in the potentiation of NGF-induced neurite outgrowth by sigma-1 receptor agonists (**Figure 8**). In addition, we found that the specific inhibitors for the Raf/Ras/MEK/MAPK pathways significantly blocked the potentiation of NGF-induced neurite outgrowth by SA4503, suggesting that these pathways are involved in the potentiation of NGF-induced neurite outgrowth by sigma-1 receptor agonists (**Figure 8**).

Our recent positron emission tomography (PET) study demonstrated that, after a single oral administration, fluvoxamine bound to sigma-1 receptors in the living human brain [45]. This finding suggests that sigma-1 receptors are involved in the mechanism of action of fluvoxamine in the human brain [45]. Taken together, the past and present findings suggest that, unlike paroxetine and sertraline, the SSRI fluvoxamine, with its sigma-1 receptor agonistic activity, might be a unique therapeutic drug for neuropsychiatric diseases.

In conclusion, the present results suggest that, as a sigma-1 receptor agonist, fluvoxamine could potentiate the NGF-induced neurite outgrowth in PC12 cells. Furthermore, it is likely that interaction with IP_3_ receptors and several subsequent signaling molecules are involved in the mechanism underlying the pharmacological action of sigma-1 receptor agonists. Therefore, it is likely that sigma-1 receptor agonists such as fluvoxamine and DHEA-sulfate, which are available around the world, would be unique therapeutic drugs for neuropsychiatric diseases.

## References

[1] Owens MJ (2004). Selectivity of antidepressants: from the monoamine hypothesis of depression to the SSRI revolution and beyond.. J Clin Psychiatry.

[2] Goodnick PJ, Goldstein BJ (1998). Selective serotonin reuptake inhibitors in affective disorders–I. Basic pharmacology.. J Psychopharmacol.

[3] Goodnick PJ, Goldstein BJ (1998). Selective serotonin reuptake inhibitors in affective disorders–II. Efficacy and quality of life.. J Psychopharmacol.

[4] Stahl SM (1998). Not so selective serotonin selective reuptake inhibitors.. J Clin Psychiatry.

[5] Stahl SM (1998). Using secondary binding properties to select a not so selective serotonin selective reuptake inhibitor.. J Clin Psychiatry.

[6] Nemeroff CB, Owens MJ (2004). : Pharmacologic differences among the SSRIs: focus on monoamine transporters and the HPA axis.. CNS Spectr.

[7] Carrasco JL, Sandner C (2005). Clinical effects of pharmacological variations in selective serotonin reuptake inhibitors: an overview.. Int J Clin Pract.

[8] Westenberg HGM, Sandner C (2006). Tolerability and safety of fluvoxamine and other antidepressants.. Int J Clin Pract.

[9] Hashimoto K, Fujita Y, Iyo M (2007). Phencyclidine-induced cognitive deficits in mice are improved by subsequent subchronic administration of fluvoxamine: role of sigma-1 receptors.. Neuropsychopharmacology.

[10] Maurice T, Urani A, Phan VL, Romieu P (2001). The interaction between neuroactive steroids and the sigma-1 receptor function: behavioral consequences and therapeutic opportunities.. Brain Res Rev.

[11] Su TP, Hayashi T (2003). Understanding the molecular mechanism of sigma-1 receptors: towards a hypothesis that sigma-1 receptors are intracellular amplifiers for signal transduction.. Curr Med Chem.

[12] Hayashi T, Su TP (2004). Sigma-1 receptor ligands: potential in the treatment of neuropsychiatric disorders.. CNS Drugs.

[13] Takebayashi M, Hayashi T, Su TP (2004). A perspective on the new mechanism of antidepressants: neuritogenesis through sigma-1 receptors.. Pharmacopsychiatry.

[14] Bermack JE, Debonnel G (2005). The role of sigma receptors in depression.. J Phamacol Sci.

[15] Hashimoto K, Ishiwata K (2006). Sigma receptor ligands: possible application as therapeutic drugs and as radiopharmaceuticals.. Curr Pharm Des.

[16] Monnet FP, Maurice T (2006). The sigma-1 protein as a target for the non-genomic effects of neuro(active)steroids: molecular, physiological, and behavioral aspects.. J Pharmacol Sci.

[17] Hayashi T, Su TP (2007). Sigma-1 receptor chaperones at the ER-mitochondrion interface regulate Ca^2+^ signaling and cell survival.. Cell.

[18] Hayashi T, Su TP (2008). An update on the development of drugs for neuropsychiatric disorders: focusing on the sigma 1 receptor ligand.. Expert Opin Ther Targets.

[19] Narita N, Hashimoto K, Tomitaka S, Minabe Y (1996). Interactions of selective serotonin reuptake inhibitors with subtypes of sigma receptors in rat brain.. Eur J Pharmacol.

[20] Okuyama S, Nakazato A (1996). NE-100: A novel sigma receptor antagonist.. CNS Drug Rev.

[21] Takebayashi M, Hayashi TP, Su TP (2002). Nerve growth factor-induced neurite sprouting in PC12 cells involves sigma-1 receptors: implications for antidepressants.. J Pharmacol Exp Ther.

[22] Hashimoto K, Scheffel U, London ED (1995). In vivo labeling of sigma receptors in mouse brain with [^3^H]4-phenyl-1-(4-phenylbutyl)piperidine.. Synapse.

[23] Takahashi H, Kirsch JR, Hashimoto K, London ED, Koehler RC (1995). PPBP [4-phenyl-1-(4-phenylbutyl) piperidine], a potent sigma-receptor ligand, decreases brain injury after transient focal ischemia in cats.. Stroke.

[24] Takahashi H, Kirsch JR, Hashimoto K, London ED, Koehler RC (1996). PPBP [4-phenyl-1-(4-phenylbutyl) piperidine] decreases brain injury after transient focal ischemia in rats.. Stroke.

[25] Harukuni I, Bhardwaj A, Shaivitz AB, DeVries AC, London ED (2000). Sigma-1 receptor ligand 4-phenyl-1-(4-phenylbutyl)-piperidine affords neuroprotection from focal ischemia with prolonged reperfusion.. Stroke.

[26] Goyagi T, Goto S, Bhardwaj A, Dawson VL, Hurn PD (2001). Neuroprotective effect of sigma-1 receptor ligand 4-phenyl-1-(4-phenylbutyl) piperidine (PPBP) is linked to reduced neuronal nitric oxide production.. Stroke.

[27] Noda Y, Kamei H, Kamei Y, Nagai T, Nishida M (2000). Neurosteroids ameliorate conditioned fear stress: an association with sigma receptors.. Neuropsychopharmacology.

[28] Maurice T, Phan VL, Urani A, Guillemain I (2001). Differential involvement of the sigma-1 receptor in the anti-amnesic effect of neuroactive steroids, as demonstrated using an in vivo antisense strategy in the mouse.. Br J Pharmacol.

[29] Matsuno K, Mita S (1998). SA 4503: A novel sigma-1 receptor agonist.. CNS Drug Rev.

[30] Matsuno K, Senda T, Kobayashi T, Okamoto K, Nakata K (1997). SA4503, a novel cognitive enhancer, with sigma-1 receptor agonistic properties.. Behav Brain Res.

[31] Gafni J, Munsch JA, Lam TH, Catlin MC, Costa LG (1997). Xestospongins: potent membrane permeable blockers of the inositol 1,4,5-trisphosphate receptor.. Neuron.

[32] Huang EJ, Reichardt LF (2001). Trk receptors: roles in neuronal signal transduction.. Annu Rev Biochem.

[33] Hayashi T, Maurice T, Su TP (2000). Ca^2+^ signaling via sigma-1 receptors: novel regulatory mechanism affecting intracellular Ca^2+^ concentration.. J Pharmacol Exp Ther.

[34] Hayashi T, Su TP (2001). Regulating ankyrin dynamics: Roles of sigma-1 receptors.. Proc Natl Acad Sci USA.

[35] Urani A, Romieu P, Portales-Casamar E, Roman FJ, Maurice T (2002). The antidepressant-like effect induced by the sigma-1 receptor agonist igmesine involves modulation of intracellular calcium mobilization.. Psychopharmacology (Berl).

[36] Chao MV (2003). Neurotrophins and their receptors: a convergence point for many signaling pathways.. Nature Rev Neurosci.

[37] Schubbert S, Shannon K, Bollag G (2007). Hyperactive Ras in developmental disorders and cancer.. Nature Rev Cancer.

[38] Sofroniew MV, Howe CL, Mobley WC (2001). Nerve growth factor signaling, neuroprotection, and neural repair.. Annu Rev Neurosci.

[39] Berridge MJ, Irvine RF (1989). Inositol phosphates and cell signalling.. Nature.

[40] Obermeier A, Bradshaw RA, Seedorf K, Choidas A, Schlessinger J, Ullrich A (1994). Neuronal differentiation signals are controlled by nerve growth factor receptor/Trk binding sites for SHC and PLC-γ.. EMBO J.

[41] Stephens RM, Loeb DM, Copeland TD, Pawson T, Greene LA (1994). Trk receptors use redundant signal transduction pathways involving SHC and PLC-gamma 1 to mediate NGF responses.. Neuron.

[42] Kimura K, Hattori S, Kabuyama Y, Shizawa Y, Takayanagi J (1994). Neurite outgrowth of PC12 cells is suppressed by wortmannin, a specific inhibitor of phosphatidylinositol 3-kinase.. J Biol Chem.

[43] Price RD, Yamaji T, Matsuoka N (2003). FK506 potentiates NGF-induced neurite outgrowth *via* the Ras/Raf/MAP kinase pathway.. Bri J Pharamcol.

[44] Price RD, Yamaji T, Yamamoto H, Higashi Y, Hanaoka K (2005). FK1706, a novel non-immunosuppressive immunophilin: neurotrophic activity and mechanism of action.. Eur J Pharmacol.

[45] Ishikawa M, Ishiwata K, Ishii K, Kimura Y, Sakata M (2007). High occupancy of sigma-1 receptors in the human brain after single oral administration of fluvoxamine: a positron emission tomography study using [^11^C]SA4503.. Biol Psychiatry.

